# Photodynamic Therapy in Ocular Oncology

**DOI:** 10.3390/biomedicines6010017

**Published:** 2018-02-07

**Authors:** Maria Antonietta Blasi, Monica Maria Pagliara, Angela Lanza, Maria Grazia Sammarco, Carmela Grazia Caputo, Gabriela Grimaldi, Andrea Scupola

**Affiliations:** Department of Ophthalmology, Catholic University, 00168 Rome, Italy; monica.pagliara@policlinicogemelli.it (M.M.P.); lanza_angela@libero.it (A.L.); mariagrazia.sam@gmail.com (M.G.S.); carmelagrazia.caputo@policlinicogemelli.it (C.G.C.); grimaldi.ga@gmail.com (G.G.); scupola99@gmail.com (A.S.)

**Keywords:** photodynamic therapy (PDT), PDT, ocular tumors, uveal melanoma, choroidal hemangioma, uveal metastases, vasoproliferative tumors

## Abstract

Although introduced for the treatment of maculopathies, photodynamic therapy (PDT) is now largely used in some eye cancers treatment. The selective tissue damage with PDT is achieved by sequestration of the photosensitizer in the target tissue and focal activation of the photosensitizer by low energy directed light. In this way, it is possible to achieve the destruction of the tumor tissue by safeguarding the surrounding healthy structures. Our study describes the clinical uses and efficacy of photodynamic therapy in ocular oncology.

## 1. Introduction

Photodynamic therapy (PDT) is a laser treatment that, through a photosensitizer like verteporfin (Visudyne^®^; Novartis International AG, Basel, Switzerland) in combination with low power and long duration infrared laser, allows for site-specific vascular occlusion and cellular destruction with minimal damage to adjacent normal structures. 

Photodynamic therapy with verteporfin causes the release of free radicals when the verteporfin is activated by laser energy. The reaction that ensues between free radicals and blood vessel endothelial cell membranes causes a local increase in immune modulation factors such as histamines, thromboxane, and TNF-α. The anti-inflammatory response can lead to a series of events including vasoconstriction, thrombosis, increased vascular permeability, blood stasis, and hypoxia. In case of neovascularization, this process serves to induce regression of the abnormal blood vessels.

After injection into the bloodstream, Visudyne (6 mg/m^2^ dose) selectively accumulates in the abnormal blood vessels in the retina and choroid. A laser beam at 689 nm (Coherent Opal Photoactivator Diode Laser; Coherent Inc., Santa Clara, CA, USA) is then applied to the lesion immediately after infusion, with a radiant exposure of 100 J/cm^2^ over an interval of 83 s, at an irradiance of 600 mW/cm^2^, over an interval of 166 s. Diode laser activates the phototoxic Visudyne sealing leaking blood vessels by triggering the release of free radicals in the areas needing treatment [1,2].

PDT was first introduced for the treatment of choroidal neovascularization from AMD, but has been extensively used in ocular oncology as well [3]. 

## 2. Photodynamic Therapy (PDT) in Choroidal Melanoma

Choroidal melanoma is the most common primary malignant intraocular tumor. The incidence rate of ocular melanoma has been reported to be 3.7% of all melanomas, with choroidal melanoma being the predominant intraocular type (86.3%), compared to less frequent iris and ciliary body melanomas [4]. Several clinical, histopathological, and cytogenetic features have been found to correlate with patients’ prognoses. In particular, the increase in pigmentation of the tumor represents a poor prognostic factor, with a 19% mortality rate at 15 years for amelanotic lesions, which is less than the 39% and 65% mortality rates reported for lightly pigmented tumors and for heavily pigmented tumors, respectively [5]. The two main treatment options for uveal melanoma patients without systemic metastasis are eye-sparing treatments and enucleation. Radiotherapy is currently the most frequently used treatment for small- and medium-size uveal melanoma, while enucleation is recommended in case of large uveal melanoma and melanomas that cause severe glaucoma or invade the optic nerve.

In clinical practice, radiotherapy can be administered as plaque brachytherapy, external beam radiotherapy, or stereotactic radiotherapy (SRT).

Plaque brachytherapy with I^125^ and Ru^106^ is currently the most common treatment modality for ocular melanoma. Brachytherapy has achieved local tumor control rates between 86% and 96% at five years, although it is associated with significant visual loss following radiation. Post-brachytherapy radiation complications such as optic neuropathy and maculopathy are determined by several factors, the most important being the total radiation dose exposure of the macula and the optic nerve. 

In a previous review, Rundle evaluated the local tumor control rate after PDT for choroidal melanoma, highlighting a local control rate ranging from 80% to 89% among series, compared with 95–97% local control rates reported for proton beam radiotherapy and stereotactic radiosurgery [6]. Despite lower control rates, the author emphasized better functional outcomes as visual acuity and the outpatient nature of this treatment modality. Nonetheless, PDT was found to be more effective in a selected study population, showing better results in patients with small melanomas. Moreover, although in some reports PDT has been effectively used for amelanotic and pigmented tumors, other authors have raised concerns as to its efficacy in treating pigmented lesions [7,8,9]. The presence of pigmentation is generally considered as an exclusion criterion for PDT because pigmentation prevents the penetration of light into the tissue. A strong correlation between the degree of regression and the degree of tumor pigmentation has indeed been found, with lighter amelanotic tumors responding much better than darker tumors to photodynamic therapy. In consideration of the strict relation between tumor thickness and total radiation dose to the macula and optic nerve, PDT has also been proposed as neo-adjuvant treatment for amelanotic choroidal melanoma, in order to reduce tumor thickness and minimize overall radiation-related toxicity. In a previous paper, we evaluated the efficacy of PDT for amelanotic choroidal melanoma, comparing visual outcomes and local control rates between patients undergoing neo-adjuvant PDT before brachytherapy versus brachytherapy alone [7]. In our study, PDT allowed for a reduction in tumor thickness in 73.4% of patients, with a minor decrease in visual acuity in pre-treated patients following radiation treatment and no local recurrence [7]. 

Another potential application of PDT, although not studied yet, is its use after brachytherapy, in the case of uveal melanomas with poor response to radiation treatment. In these cases, it has been reported that, following photodynamic treatment, the tumor showed important and rapid regression. A possible explanation could be that, after brachytherapy, together with the reduction in tumor mass, a recirculation and re-oxygenation process occurs. This would improve the concentration of verteporfin in tumor vessels and thus the melanoma sensitivity to the treatment [10].

## 3. PDT in Choroidal Hemangioma

Circumscribed choroidal hemangioma (CCH) is a benign tumor. In most cases, it is a lone and circumscribed lesion but can also be found in association with Sturge-Weber syndrome. It manifests as a newly discovered, dome-shaped, orange-red mass, typically located at the posterior pole. When symptomatic, it may cause a variable reduction in visual acuity [11]. Treatment is necessary when the patient’s vision is decreased or is threatened due to secondary exudative retinal detachment, macular edema, subfoveal fluid, or in case of potential central vision impairment due to juxtafoveal tumor [12]. Xenon arc, argon laser photocoagulation, transpupillary thermotherapy (TTT), and radiotherapy have been reported in the literature as treatments of such lesions. However, these treatments have limited efficacy because of the risk of foveal scarring in the case of central lesions, limiting the visual outcome [13]. Radiation-induced complications also limit the use of radiation therapies [14]. 

Photodynamic therapy (PDT) with verteporfin has been shown to be an effective treatment for CCH, with the additional benefit of selectively preserving the overlying neuroretinal structures [15,16,17,18].

Recently, a prospective, nonrandomized, multicenter clinical trial enrolled 31 patients to study the effect of PDT on patients with CCH [17]. A decrease in CCH thickness and a resolution of exudative detachment were observed in all cases, with visual recovery in 69% of patients. Patients with symptomatic juxafoveal CCH who were treated with PDT have shown tumor regression, preservation of the overlying retina, and either stable or improved visual acuity. 

In our prospective study, we evaluated PDT efficacy for symptomatic untreated CCHs over five years of follow-up in the largest case series to date of patients receiving PDT as a primary treatment. All patients have shown an excellent response to PDT, with rapid reabsorption of subretinal fluid and flattening of the hemangioma. This result can be related to the selective damage to endothelial cells induced by PDT, generating selective vascular occlusion with minimal damage to overlying retinal structures [19]. 

Repeated PDT sessions can be performed if inadequate tumor regression is noted, but delayed choroidal atrophy can result from repeated sessions on previously treated areas or overlapping exposure [20]. 

## 4. PDT in Vasoproliferative Tumor

Vasoproliferative tumor (VPT) is a benign retinal tumor that appears as a reddish-pink, nodular mass located in the pre-equatorial region with mildly dilated retinal feeder vessels. It often causes vision-threatening complications as lipid exudation, cystoid macular edema (CME), vitreous hemorrhage, retinal detachment, and ERM formation. It can be primary (idiopathic, 80%) or secondary (associated) to other ocular diseases such as retinitis pigmentosa, sickle cell retinopathy, Coat’s disease, retinopathy of prematurity, toxoplasmosis, toxocariasis, tuberculosis, other forms of uveitis, ocular trauma, retinalchoroidal coloboma, and retinal detachment [21,22,23].

VPT usually presents as a solitary mass in the retinal periphery. The inferior retina is affected in 60–90% of cases and the temporal retina in 42–75% of cases. VPTs may exhibit feeder vessels, although with less dilation and tortuosity than those seen in retinal capillary hemangioma, which is its major differential diagnosis. Despite its benign nature, VPT can cause severe visual loss due to secondary involvement of the vitreous and retina (epiretinal and subretinal membranes, vitreous hemorrhage, subretinal fluid, and exudation) or, less frequently, neovascular glaucoma. 

Management options include observation of peripheral smaller tumors with minimal exudation posing no visual threat. Tumors <2 mm may be treated with laser photocoagulation or cryotherapy. 

Larger VPT (>2 mm) can be treated with plaque radiotherapy (ruthenium^106^ or iodine^125^) with tumor regression in >90% and resolution of exudation and subretinal fluid [24].

PDT has been reported as an effective treatment in retinal and choroidal vascular tumors [25,26]. There are a few reports of its successful use in VPT even for larger tumors, like the ones reported in our experience, with a thickness varying from 2.03 to 4.45 mm [27]. Its major limitation is the technical difficulty to reach the typical peripheral location of VPTs. Although other treatment modalities may be more effective in eradicating the tumor, PDT provides a minimally invasive, easily accessible treatment with no apparent side effects. 

## 5. PDT in Choroidal Metastasis

Choroidal metastases are the most common intraocular malignancy in adults. Uveal metastases most frequently originate from primary tumors of the breast (47%), lung (21%), gastro intestinal tract (4%), kidney (2%), skin (2%), prostate (2%), and other locations (4%). In about 17% of patients the primary tumor remains unknown. Symptoms due to ocular metastases depend on the location and characteristics of the lesions. Although most patients remain asymptomatic [28], blurred vision and a decrease in visual acuity can be observed, with a negative impact on the quality of life of affected patients [29]. These symptoms are due to the posterior localization of metastasis and the associated exudation [30]. Other symptoms include visual field scotoma, floaters, metamorphopsia, photopsia, soreness, and visual flashes [31,32].

Management of ocular metastases is palliative since treatment is limited to the primary tumor. 

Despite improvements in systemic treatments of primary malignancy such as breast and lung adenocarcinoma, patients with uveal metastasis have a high mortality. Patients with uveal metastasis from breast cancer show survival rates of 65% at one year, 35% at three years, and 24% at five years. In uveal metastasis from lung cancer, there is tumor-related death at one year in 54% of patients. Particularly in patients with limited survival, the morbidity and duration of the treatment itself must be considered [33] and the goal of local treatments is aimed at restoring visual function, minimizing ocular toxicity [29]. 

The actual benefit of a local treatment should be considered in view of the limited life expectancy of metastatic patients. Therefore, it is important to provide an acceptable quality of life by improving the visual condition of affected patients.

There are several cases of effective PDT treatment of metastases [34,35,36,37]: Except for two cases, all patients responded with tumor size decrease. Kaliki et al. reported a small case series of eight patients with nine choroidal metastases treated with PDT with a good response in 82% of cases [37].
